# A study that redefines the concept of a well-known orchid–pollinator attraction strategy

**DOI:** 10.1093/nsr/nwae129

**Published:** 2024-04-01

**Authors:** Hong Liu

**Affiliations:** Department of Earth and Environment, International Center for Tropical Botany, Florida International University, USA; The Fairchild Tropical Botanic Garden, USA

Zhang and collaborators [1] demonstrated that bees of the genus *Ctenoplectra* collect oil from the newly reported oil-reward orchids of the genera *Dendrobium* and *Galeola* in Asia, and that the majority of the bees that carry out this act and pollinate the orchids are male. These are new discoveries with significant implications for biodiversity conservation.

In-depth knowledge on the interaction web of species is critical in formulating effective conservation strategies for species that are in danger of extinction, as is the case for a neotropical oil-reward mimic orchid [2]. While we know that species interactions can be complex, the discovery of Zhang *et al.* reminds us that we are still far from knowing the true nature of these interaction webs.

Orchid–pollinator interactions are probably one of the best systems for illustrating the complexity and intrigues of nature's interaction web. Globally, orchid pollinators are highly diverse with vastly varied life histories [3]. An example of the most fascinating, specialized orchid–pollinator interactions is the male euglossine bees in tropical America, which collect particular plant chemicals from perfume orchids to be presented to female bees as part of the courtship. Both male and female euglossine bees would collect nectar and pollen from other plants to fulfill their nutritional needs [4]. Conservation of the perfume orchids would demand the protection of the pollinating bees as well as other species in this interaction web.

In another equally specialized and complex orchid–pollinator relationship, oil-reward offering or mimicking orchids in tropical America are visited and pollinated by oil-collecting bees. This pollinator attraction strategy involving floral oil rewards is currently defined based on the most comprehensive global orchid–pollinator database, as ‘(orchids offered floral) lipids (that) are collected by FEMALE bees to construct or provision nest cells’ [3]. The discovery by Zhang *et al.* [1] that male bees of the genus *Ctenoplectra* collect oil from oil-offering orchids of the Asian genera *Dendrobium* and *Galeola* demands the revision of this well-accepted concept of the lipid-reward pollinator attracting strategy to include male bees!

The purpose of the oils collected by the *Ctenoplectra* females is assumed to be as food for broods, as are the oils collected by the female *Centris* bees in tropical America. Zhang *et al.* [1] showed that the male *Ctenoplectra* bees would transfer the oil to the females during mating. Therefore, oils collected by the male *Ctenoplectra* are likely to be copulatory ‘gifts’ for the female bees. If this is proven to be the case (more work is needed here), then *Ctenoplectra* becomes one of the many insect species that engage in the romantic behavior of copulatory gifting.

**Figure 1.**
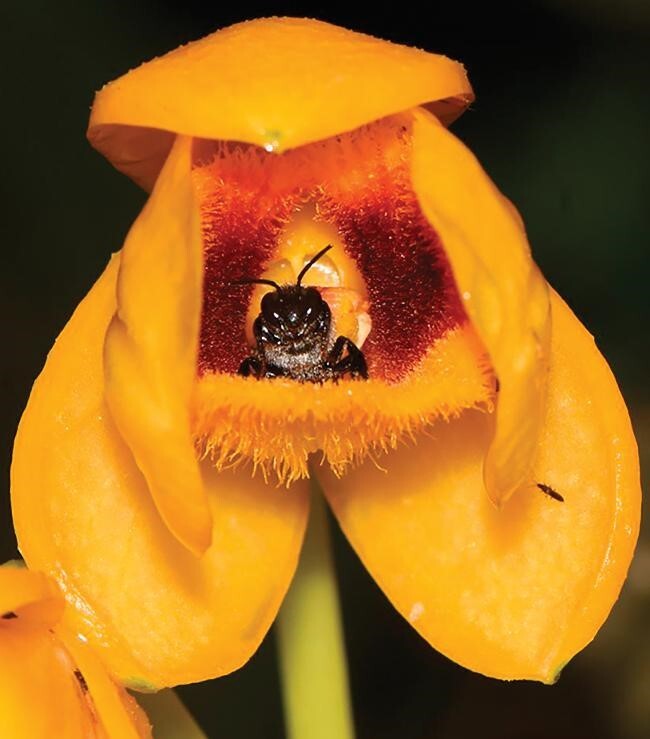
Male *Ctenoplectra cornuta* bee collecting oil secretions from *Dendrobium chrysanthum* labellum in southern Yunnan, China, photographed by Meng Zhang.

The report of oil secretion in nearly 79.5% (*n* = 39) of the *Dendrobium* species studied (see Tables S1 and S2 in [1]) is also the first to discover oil-offering orchids that are exclusively pollinated by oil-collecting bees in Asia (also see [5] on oil-offering *Cymbidium aloifolium* pollinated by a generalist honeybee *Apis cerana*). These discoveries may indicate that the orchid-oil-reward flower system could involve a large number of orchids in Asia, as there are 1647 species in the genus (https://www.worldfloraonline.org/taxon/wfo-4000011060). *Dendrobium* has many species of horticultural and medicinal significance, and a more thorough understanding of the ecology of these species would help in the sustainable use and conservation of these valuable biological resources. Zhang *et al.* [1] represents a major contribution to our knowledge of orchid pollination in tropical Asia and globally (Fig. 1).
